# Prevalence and genotypes distribution of virus hepatitis B and hepatitis delta virus in chronic liver diseases in Kazakhstan

**DOI:** 10.1186/s12879-023-08524-1

**Published:** 2023-08-14

**Authors:** Bibigul S. Ilyassova, Balzhan Abzhaparova, Dariga S. Smailova, Aidos Bolatov, Bolatbek Baymakhanov, Vyacheslav Beloussov, Maxim Solomadin, Kunsulu Shamsivaliyeva, Gulnara Alpysbayava, Gaukhar Issakova, Joanna Granica, Dina Mukushkina, Inkar Y. Sagatov, Shokan Kaniyev

**Affiliations:** 1 JSC “National Scientific center of Surgery named after A.N.Syzganov”, Zheltoksan str. 62, Almaty, 050004 Kazakhstan; 2 Asfendiyarov Kazakh National Medical University, Almaty, Kazakhstan; 3 Astana Medical University, Astana, Kazakhstan; 4 Molecular Genetics Laboratory “TreeGene”, Almaty, Kazakhstan; 5 Karaganda Medical University, Karaganda, Kazakhstan

**Keywords:** Hepatitis B virus, Hepatitis D virus, delta agent, Genotype, Phylogenetic analysis, PCR sequencing

## Abstract

**Background:**

The geographical distribution of hepatitis B virus (HBV) and hepatitis D virus (HDV) genotypes is uneven and has its own clinical and organizational implications for health systems. Despite the introduction of vaccination and successful antiviral therapy the prevalence of chronic hepatitis B (with or without delta agent) increased over the past 5 years. This study aimed for the first time to investigate the molecular epidemiology of HBV and HDV in Kazakhstan.

**Methods:**

Total 834 chronic hepatitis B (with or without delta agent) patients were included to the study from November 2017 to June 2019. The material was collected from the regional hepatological сenters from 13 cities of Kazakhstan. Genotyping of HBV/HDV isolates was carried out using phylogenetic analysis of null-binary sequences of Kazakhstani isolates, in comparison with the reference sequences. Nucleotide sequence alignment was performed using the ClustalW algorithm, the “neighbor-joining” method was used for the construction of phylogenetic trees and subsequent analysis.

**Results:**

Overall 341 samples were PCR-positive and genotyped for HBV. Comparison and phylogenetic analysis of nucleotide sequences of HBV isolates showed that they were represented by genotypes HBV-D (95.9%), HBV-A (3.5%) and HBV-C (0.6%). At the same time, the identity of the nucleotide sequences of Kazakhstani isolates were: HBV-D (95–100%); HBV-A (97.2–100%) and HBV-C (99%). 256 samples were PCR positive and genotyped for HDV, all of them belonged to genotype 1.

**Conclusion:**

This study describes for the first time the molecular epidemiology of HBV and HDV in Kazakhstan. The data obtained expand the knowledge of the global epidemiology of viruses; have potential implications for public health policy and for further clinical research on chronic hepatitis in Kazakhstan.

**Trial registration:**

ClinicalTrials.gov NCT05095181 (registered on 27/10/2021).

**Supplementary Information:**

The online version contains supplementary material available at 10.1186/s12879-023-08524-1.

## Introduction

The burden of hepatitis B infection is highest in the WHO Western Pacific Region and the WHO African Region, where 116 million and 81 million people, respectively, are chronically infected. Sixty million people are infected in the WHO Eastern Mediterranean Region, 18 million in the WHO South-East Asia Region, 14 million in the WHO European Region and 5 million in the WHO Region of the America [1]. Hepatitis D, caused by the hepatitis Delta virus (HDV) is a unique RNA pathogen, which needs hepatitis B virus (HBV) envelope proteins to infect the hepatocytes [2, 3]. The level of HBV infection in Kazakhstan is high, affecting approximately 8% of the population [4]. During the 2014–2019, prevalence, incidence and mortality from chronic HBV infections increased in Kazakhstan [5]. According to the Data of the Unified National Electronic Health System (UNEHS) for the period 2014–2019, 35,881 patients with chronic hepatitis B were registered in Kazakhstan, among which 10% were with a delta agent [6]. Despite the undoubted progress achieved through the introduction of vaccination and successful antiviral therapy, the number of patients with chronic viral hepatitis B in Kazakhstan in 2021 was 4792 adults, with chronic viral hepatitis B with a delta agent 1239 people. Moreover, from 2015 to 2020, the prevalence of hepatitis B increased from 64.3 to 128.3 cases per 100,000 populations, while the prevalence of hepatitis D increased from 4.13 to 13.03 cases per 100,000 populations over the same period [7].

Superinfection of HDV in individuals with HBV infection leads to progressive diseases and cirrhosis in approximately 80% of cases. Cirrhosis develops at a younger age than in patients with chronic HBV infection only [8]. In 10–15% of cases, cirrhosis develops in 2 years; 70–80% of cirrhosis develops in 5–10 years with HBV with delta agent. The risk of developing HCC is 2.9-6.0 times higher in patients with superinfection than in patients with HBV monoinfection [8, 9]. HBV co-infection with HDV is significantly associated with development at a younger age than with monoinfection [10]. HBV with delta agent remains the most common cause of liver transplantation in Kazakhstan. During the period from 2012 to 2021, among all 207 liver transplantations in A.Syzganov’s National Scientific Center of Surgery (Almaty, Kazakhstan), 96 (39.1%) were due to HBV with delta agent.

The course of HBV infection and progression of chronic HDV infection may depend on host and viral factors [7, 11]. Important factor of progression and clinical features are the HBV and HDV genotypes. The prognosis of the disease depends on the characteristics of the HDV and HBV genotypes in CHB with delta agent. Thus, genotyping of HBV and HDV have clinical and epidemiological significance [8–11]. However, there is no data on the prevalence of the HBV and HDV genotypes in Kazakhstan. Consequently, study aimed at evaluating the genotypic structure of HBV and HDV in patients with chronic viral hepatitis in Kazakhstan.

## Materials and methods

### Study population

The study involved 834 adults with chronic hepatitis B (the presence of HBsAg in the blood or serum for more than 6 months), who were treated in regional hepatological centers during the November 2017 to June 2019 in 13 cities of Kazakhstan.

### Ethics statement

Study was approved by the institutional review board of Local Ethical Committee of the National Scientific Center of Surgery named after A.N. Syzganov (Almaty, Kazakhstan) on 14 September 2017. Informed written consent was obtained after detailed explanation of the study at the time of sampling from all participants.

### Serological analysis

HbsAg (Abbott ARCHITECT^®^, USA) and anti-HDVAg (Vector-Best, Russia) were detected by commercial ELISA tests.

### DNA isolation

Venous blood from HBV and HDV positive patients were used for studies. HBV DNA and HDV RNA were isolated using the GeneJET Viral DNA/RNA Purification Kit (Thermo Fisher Scientific™, USA) according to the manufacturer’s instructions. 200 µl of venous blood with EDTA was used for isolation. cDNA from RNA was obtained using the High-Capacity cDNA Reverse Transcription Kit (Applied Biosystems™, USA) according to the manufacturer’s instructions. DNA/cDNA was stored at -20^0^ C.

### PCR for DNA amplification

PCR was performed using a set of specific primers proposed by Sitnik et al. (2004) for HBV and Shahinsaz et al. (Shahinsaz et al., 2006) for HDV, the characteristics of which presented in Tables S1 and S2 [12, 13]. The selected primers corresponded to conserved regions among different genotypes, flanking heterogeneous regions to distinguish HBV and HDV genotypes.

In the absence of a PCR product of a given size, a second round of PCR was performed with nested primers and under the same conditions, instead of DNA, PCR products of the first round were used. Positive and negative control samples were used during PCR. Both rounds of PCR were carried out with a total volume of the reaction mixture of 50 µl.

Amplification of PCR products was carried out on a GeneAmp® PCR System 9700 thermal cycler (Applied Biosystems™, USA). Detection and separation of PCR products was carried out in 2% agarose gel, on TAE buffer, using GeneRuler 100 bps DNA Ladder (Thermo Fisher Scientific™, USA). Gels were stained using UltraPure™ Ethidium Bromide (Invitrogen™, USA). Samples with specific PCR products were further used for purification and subsequent sequencing.

### Sequencing of PCR products and nucleotide sequence analysis of HBV and HDV

Before sequencing, PCR products of HBV/HDV were purified from primer and nucleotide residues using ExoSAP-IT™ PCR Product Cleanup Reagent (Applied Biosystems™, USA) according to the manufacturer’s instructions. The purified PCR products were sequenced in two directions, using a forward and reverse FHBS1-RHBS1 or FHBS2-RHBS2 primers for HBV and HD-1,2 or HD-3,4 primers for HDV (developed by ThermoFisher ScientificTM, USA), in accordance with the manufacturer’s instructions for BigDye Terminator v3.1 Cycle Sequencing Kit (Applied Biosystems™, USA).

Electrophoresis and separation of labeled fragments were performed on a 3130 Genetic Analyzer (Applied Biosystems™, USA). Before electrophoresis, the PCR mixtures were cleaned of terminator residues using BigDye XTerminator (Applied Biosystems™, USA), in accordance with the manufacturer’s instructions. The analysis of the primary data was carried out using the Sequencing Analysis Software program (Applied Biosystems™, USA). The alignment and assembly of HBV and HDV nucleotide sequences was performed using the DNA Baser Sequence Assembly Software program.

### Genotyping and HBV/HDV reference sequences

Verification of HBV DNA and HDV RNA sequences was carried out using the Nucleotide BLAST application as well as using the Genotyping (HBV) application [14]. HBV/HDV reference sequences for genotyping and phylogenetic analysis were obtained from GeneBank, the National Center for Biotechnology Information and the U.S. National Library of Medicine and presented in Table S3.

Genotyping of HBV/HDV isolates was carried out using phylogenetic analysis of null-binary sequences of Kazakhstani isolates, in comparison with the reference sequences indicated above and the data obtained during Nucleotide BLAST. Nucleotide sequence alignment was performed using the ClustalW algorithm of the Molecular Evolutionary Genetic Analysis (MEGA) version 7, and for the construction of phylogenetic trees and subsequent analysis, the method “neighbor-joining (NJ)” was used [15, 16].

## Results

Among 834 patients registered in hepatological centers of the Republic of Kazakhstan, 487 samples were positive for HBsAg at the time of the study, among which 341 were PCR-positive for HBV and genotyped. Among 487 HBsAg-positive samples, 261 samples were also positive for HDAg, while 256 were PCR-positive for HDV and genotyped.

### Hepatitis B virus genotyping results

Patients’ blood (487 samples in total) positive for HBsAg were obtained from the following regions of the Republic of Kazakhstan: Aktau, Atyrau, Oskemen, Turkestan, Taraz, Chimkent, Almaty, Astana, Kyzylorda, Pavlodar, Aktobe, Kostanay and Karaganda.

In total, out of 487 samples, 341 samples were positive for PCR and genotyped (70% of the total number of submitted samples). Comparison and phylogenetic analysis of nucleotide sequences of PCR products of HBV isolates showed that they (n = 341) are represented by genotypes HBV-D (95.9%; n = 327), HBV-A (3.5%; n = 12) and HBV-C (0.6%; n = 2). At the same time, the identity of the nucleotide sequences of Kazakhstani isolates was: HBV-D (95–100%; n = 327); HBV-A (97.2–100%; n = 12) and HBV-C (99%; n = 2) (Table 1).

**Table 1:** Data on genotyping of HBV isolates isolated in the territory of the Republic of Kazakhstan, by region

Region	Genotype		
	HBV-D	HBV-A	HBV-С
Aktau	20	0	0
Atyrau	22	0	0
Oskemen	36	0	0
Turkestan	32	1	0
Taraz	16	2	0
Shymkent	67	3	0
Almaty	25	4	2
Astana	6	0	0
Kyzylorda	18	1	0
Pavlodar	30	1	0
Kostanay	24	0	0
Karaganda	20	0	0
**Total Quantity**	327	12	2
**Occurrence, %**	95.9%	3.5%	0.6%
**Identity, %**	95.0-100%	97.2–100%	99.7%

The most common HBV genotype in Kazakhstan was the D genotype with a frequency of 95.9% of the total number of sequenced samples. This genotype met and prevailed in all the studied regions. Genotype A was found in 3.5% of cases, and was found in the following regions of the Republic of Kazakhstan: Turkestan, Taraz, Chimkent, Almaty, Kyzylorda and Pavlodar, mostly in the southern regions RK. Genotype C was found only in two cases, and these were samples from Almaty. Previously, the presence of this genotype in the territory of the Republic of Kazakhstan was not reported in the literature. In 21 patients with positive PCR of HBV DNA and HDV RNA, hepatitis B virus genotype D was detected.

**Fig. 1:**
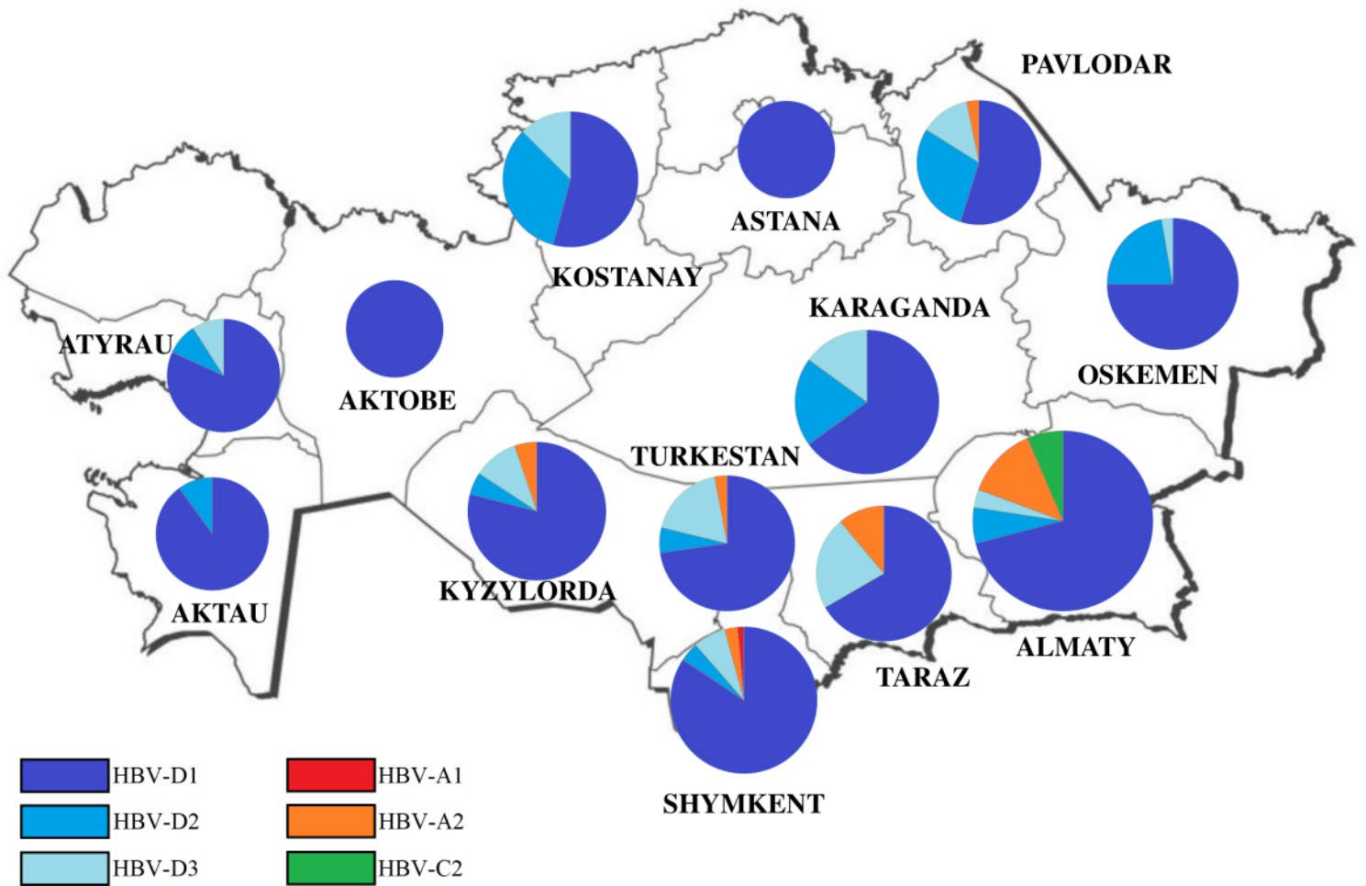
The prevalence of hepatitis B virus subgenotypes in the regions of Kazakhstan

**Fig. 2:**
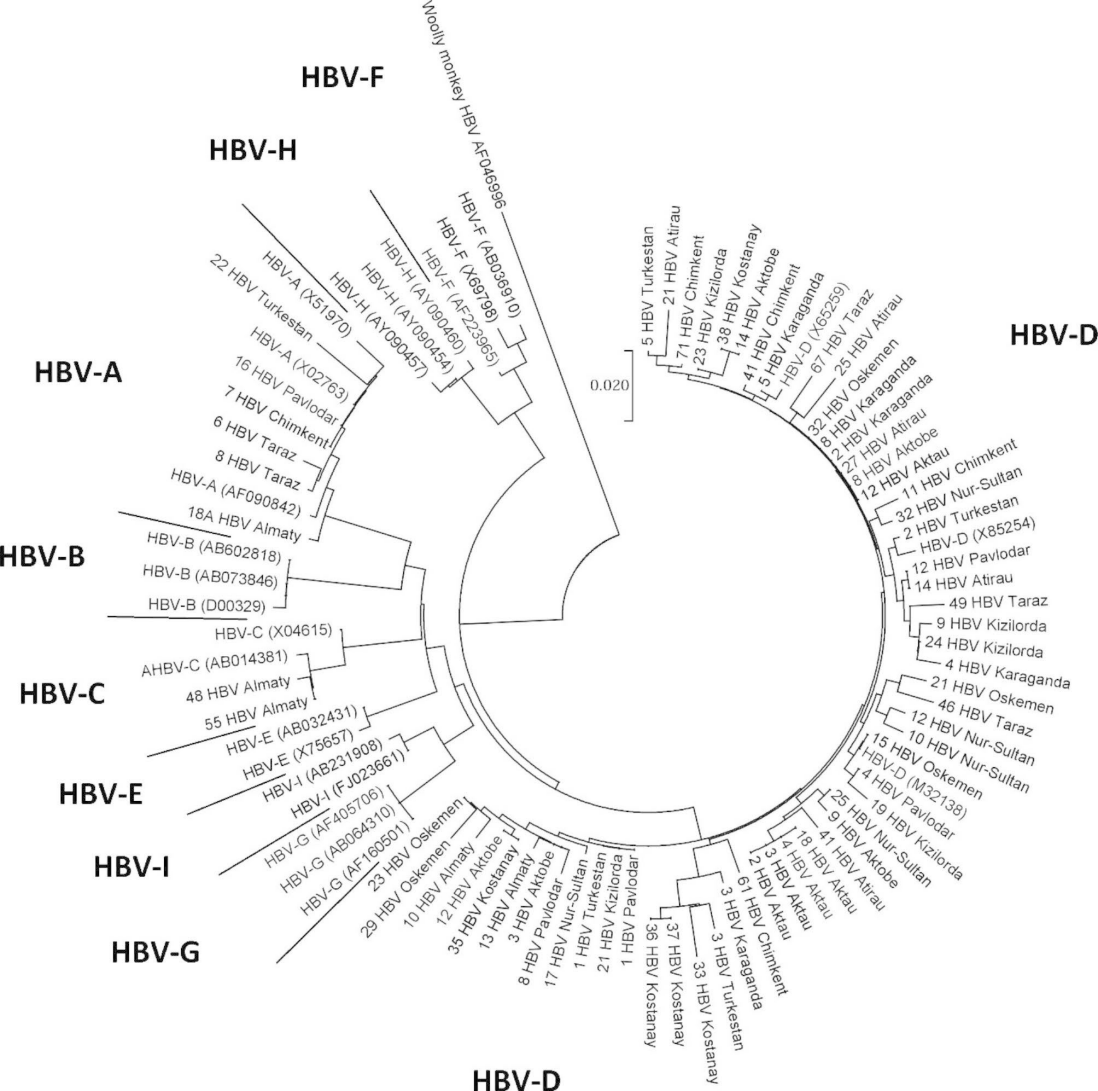
Phylogenetic tree for HBV in Kazakhstan

In current study the most common subgenotype was D1–61.5% (Fig. 1). Among patients with HDV, both HBV DNA and HDV RNA (circulation of both viruses) were determined in 37 studies of genotypes and subgenotypes of hepatitis B virus showed that 32 patients (86.5%) have genotype D1, 2 patients - A2 (5.4%), 1 patient - D1 (2.7%), 1 - D3 (2.7%), 1 - D1 + D3 (2.7%). Phylogenetic trees for HBV presented in Fig. 2.

### The result of a study of hepatitis D virus genotypes

Patient blood (261 samples) positive for HBsAg and anti-HDVAg ELISA were received from the following regions: Taraz, Chimkent, Almaty and Almaty region, Kyzyl-Orda, Pavlodar, Aktobe, Oskemen, Aktau, Atyrau, South Kazakhstan region.

**Fig. 3:**
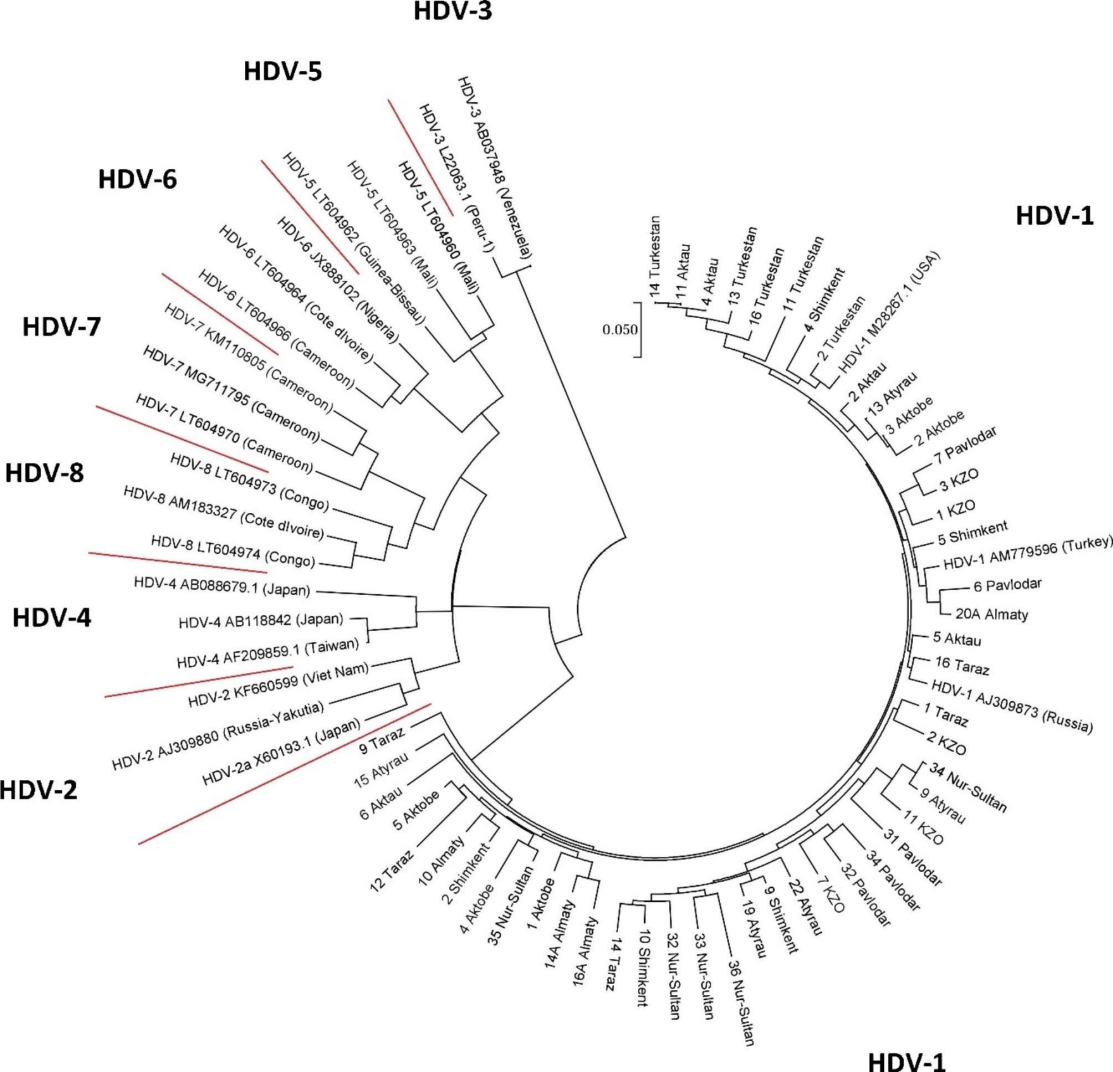
Phylogenetic analysis of HDV isolates from the Republic of Kazakhstan, together with isolates and strains of the virus, presented in the international GenBank database. All Kazakhstani isolates are included in one common HDV-1 cluster

In total, out of 261 samples, 256 samples (98%) were PCR positive and genotyped. Comparison and phylogenetic analysis of the nucleotide sequences of PCR products of HDV isolates showed that they all belonged to genotype 1 (Fig. 3), which is consistent with the literature data on the spread of the virus in the world. At the same time, the identity of the nucleotide sequences isolated on the territory of the Republic of Kazakhstan was 79.1–99.7%.

## Discussion

This study described for the first time the molecular (genetic) epidemiology of hepatitis B and hepatitis Delta viruses in Kazakhstan. A total sample of 341 people with HBV and 256 people with HDV were genotyped.

The most common HBV genotype was the D genotype (95.9%), specifically subgenotype D1 (61.5%) in Kazakhstan. However, genotype D is prevalent in Africa, Europe, the Mediterranean region and India, and genotypes B and C are common in Asia [17–19]. At the same time, recent data indicate that subgenotype D1 is widely distributed in all continents, but is highly prevalent in Asia. Thus, in the countries bordering Kazakhstan, genotype D highly prevalent in Kyrgyzstan – 100% (in 30 cases), in Russia – 89% (in 471 cases), and in Uzbekistan – 83% (in 118 cases). These countries had deep historical and cultural relationships with Kazakhstan. Whereas, in China, which also has a common border with Kazakhstan, genotypes B and C were more common [20]. It is known that genotype D has a higher risk of transition to a chronic infection and a high precore A1896 mutation frequency which associated with more aggressive liver diseases [20, 21]. In confirmation more than 90% of patients had chronic viral hepatitis B associated with genotype D in Kazakhstan. Moreover, among patients with chronic hepatitis B with delta agent, the highest frequency of occurrence of the subgenotype D1 (61.5%) was noted; whereas other subgenotypes were less common (D2 in 10.6%, D3 in 9.6% of cases).

In addition to genotype D of the HBV, genotype A (7.7%) and Genotype C (1.0%) were detected. In some regions of Kazakhstan (Pavlodar, Almaty, Zhambyl and Kyzylorda), HBV genotype A were identified, which has better clinical outcomes and a higher response to interferon alpha [18]. Compare to other regions (besides Pavlodar), HBV-A genotype were identified in the southern regions of Kazakhstan (Kyzylorda, Turkestan, Shymkent, Taraz, Almaty), this is most likely due to the high migration status of these regions with Uzbekistan, in which the occurrence of this genotype is about 15% [22]. We hypothesize that the presence of all three HBV-D,A,C genotypes (determined in Kazakhstan) in Almaty is due to several factors, including: (1) until 1997, Almaty was the capital of Kazakhstan and had the highest migration load; (2) this region is the most touristic in Kazakhstan; (3) and it is no less important that this region is both geographically and economically close to China, where the frequency of the genotype C is 64% [22].

Genotyping of the HDV revealed the presence of only genotype HDV-1 in the population of Kazakhstan. Genotype HDV-1 is the most common and it is present worldwide [23–25]. Moreover, HDV-1 showed the highest pathogenic potential compare to other genotypes [23].

## Conclusion

Current study was the first to describe the molecular epidemiology of HBV and HDV in Kazakhstan. The genotype D (namely, the D1 subgenotype) of the HBV was predominant, and in all cases, the genotype 1 of the HDV was identified. The data obtained expand the knowledge of the global epidemiology of HBV and HDV and have clinical and organizational implications for the healthcare system in Kazakhstan.

### Electronic supplementary material

Below is the link to the electronic supplementary material.

Supplementary Material 1

## Data Availability

All data available by request to corresponding author.

## Declarations

### Clinical trial registration

ClinicalTrials.gov NCT05095181 (registered on 27/10/2021).

## List of abbreviations

HBV: Hepatitis B virus
HDV: Hepatitis D virus
WHO: World Health Organization
CHB: Chronic hepatitis B
