# Supplementary material for: Prevalence and genotypes distribution of virus hepatitis B and hepatitis delta virus in chronic liver diseases in Kazakhstan
Source: BMC Infect Dis. 2023 Aug 14;23:533. doi: 10.1186/s12879-023-08524-1 (PMC10426108; doi:10.1186/s12879-023-08524-1)
Supplement: Supplementary file 1 — Supplementary Material 1 [file 12879_2023_8524_MOESM1_ESM.docx]

Supplementary materials

Table S1 - Primers used in studies to amplify the HBV S-gene region.

| **Name** | **Subsequence, 5’-3’** | **Position** | **PCR product size** |
| --- | --- | --- | --- |
| FHBS1 | GAG TCT AGA CTC GTG GTG GAC TTC | 244-267 | 447 bps |
| RHBS1 | AAA TKG CAC TAG TAA ACT GAG CCA | 668 to 691 |  |
| FHBS2 | CGT GGT GGA CTT CTC TCA ATT TTC | 255-278 | 416 bps |
| RHBS2 | GCC ARG AGA AAC GGR CTG AGG CCC | 648-671 |  |

Table S2 - Primers used in studies to amplify the C-terminal region of HDAgHDV.

| **Name** | **Subsequence, 5’-3’** | **Position** | **PCR product size** |
| --- | --- | --- | --- |
| HD1 | CCA GGT CGG ACC GCG AGG AGG | 855-872 | 452 bp |
| HD2 | ACA AGG AGA GGC AGG ATC ACC GAC | 1284-1307 |  |
| HD3 | GAT GCC ATG CCG ACC CGA AGA | 880-901 | 400 bp |
| HD4 | GAA GGA AGG CCC TCG AGA ACA AGA | 1260-1280 |  |

Table S3. HBV/HDV reference sequences for genotyping and phylogenetic analysis.

| HBV | HDV |
| --- | --- |
| HBV-A–AF090842, X02763, X51970; HBV-B –AB073846, AB602818, D00329; HBV-C –AB014381, X04615; HBV-D – M32138, X65259, X85254; HBV-E – AB032431, X75657; HBV-F – AB036910, AF223965, X69798; HBV-G– AB064310, AF160501, AF405706; HBV-H – AY090454, AY090457, AY090460; HBV-I - AB231908, FJ023661.  Sub-genotypes: HBV-D1 - FJ437079 (Iran), JX090646 (Russia), JX090687 (Russia), JX090723 (Russia), JX090724 (Russia), KT201311 (Pakistan), KY210472 (Uzbekistan), KY210473 (Uzbekistan), KY210482 (Uzbekistan), KY629630 (China),KP191647 (Arabian); HBV-D2–EU594402 (Estonia), EU594409 (Estonia), EU594422 (Russia), EU594429 (Russia), GQ477452 (Poland), JX096958 (Latvia), KX372183 (Belgium), KX827290 (USA), KY810020 (Brazil), KY816238 (Belarus); HBV-D3–JX090608 (Russia), JX090612 (Russia), JX090709 (Russia), KY816260 (Belarus), KY816265 (Belarus), KY816275 (Belarus), KY816277 (Belarus);HBV-D4 - AB033559 (Papua), AB048701 (Australia), FJ692532 (Haiti), FJ692533 (Haiti), FJ692536 (Haiti);HBV-D5–AB033558 (Japan), GQ205377 (India), GQ205382 (India), GQ205384 (India), GQ205389 (India);HBV-D6–FJ904394 (Tunisia), FJ904395 (Tunisia), FJ904433 (Tunisia), FJ904438 (Tunisia), FJ904441 (Tunisia);HBV-A1 – AB116087 (India), AB116088 (Nepal), AB453988 (Japan), FJ692592 (Haiti), AF090842 (Belgium);HBV-A2 – AB014370 (Japan), AY128092 (Canada), Z35717 (Poland), Z72478 (Germany), AJ344115.1 (France);HBV-A3 – AM180624 (Cameroon), AY934763 (Gambia), AY934764 (Gambia);HBV-A4 – GQ331048 (Belgium);HBV-C1 – AB074756 (Thailand), AB112063 (VietNam), AB112471 (Thailand), AB112472 (Thailand), AB117758 (Cambodia), AF068756 (Thailand);HBV-C2 – AB014362 (Japan), AB014376 (Japan), AB205123 (China), AY066028.1 (China);HBV-C3 – X75656 (Polynesia), X75665 (NewCaledonia);HBV-C4 – AB048704 (Australia), AB048705 (Australia);HBV-C5– AB241109(Philippines), AB241110 (Philippines), AB241111 (Philippines);HBV-C6 – AB493842 (Indonesia), AB554014 (Indonesia); HBV-C8 – AP011104 (Indonesia), AP011107 (Indonesia). | HDV1 - AM779596(Turkey), AJ309873(Russia), M28267.1(USA); HDV2 - KF660599 (VietNam), LT604953 (Russia), AJ309880 (Russia-Yakutia); HDV3 - AB037948 (Venezuela), KF786343 (Brazil), L22063.1 (Peru-1); HDV4 - AB088679.1 (Japan), AB118842 (Japan), AF209859.1 (Taiwan); HDV5 - LT604960 (Mali), LT604962 (Guinea-Bissau), LT604963 (Mali); HDV6 - JX888102 (Nigeria), LT604964 (Coted'Ivoire), LT604966 (Cameroon); HDV7 - KM110805 (Cameroon), LT604970 (Cameroon), MG711795 (Cameroon); HDV8 - AM183327 (Coted'Ivoire), LT604973 (Congo), LT604974 (Congo). |
